# The Prevalence of Lymphatic Filariasis in Elementary School Children Living in Endemic Areas: A Baseline Survey Prior to Mass Drug Administration in Pekalongan District-Indonesia

**Published:** 2018-10

**Authors:** Praba GINANDJAR, Lintang Dian SARASWATI, Dedy SUPARYANTO, Mateus SAKUNDARNO, Taniawati SUPALI

**Affiliations:** 1. Dept. of Epidemiology, Faculty of Public Health, Diponegoro University, Semarang, Indonesia; 2. Doctoral Program of Medical and Health Sciences, Faculty of Medicine, Diponegoro University, Semarang, Indonesia; 3. Master Program of Biomedical Sciences, Faculty of Medicine, Diponegoro University, Semarang, Indonesia; 4. District Health Office, Pekalongan District, Central Java, Indonesia; 5. Master Program of Epidemiology, School of Postgraduate Studies, Diponegoro University, Semarang, Indonesia; 6. Dept. of Parasitology, Faculty of Medicine, University of Indonesia, Jakarta, Indonesia

**Keywords:** Mass treatment, *Wuchereria bancrofti*, Elimination, Filariasis, Children

## Abstract

**Background:**

WHO initiated lymphatic filariasis (LF) elimination globally. Pekalongan District, as LF endemic area, started a program of mass drug administration (MDA) to combat LF in 2015. This study aimed to determine prevalence of *Wuchereria bancrofti* infection prior to the MDA.

**Methods:**

LF infection was detected by the existence of circulating filarial antigen (CFA) *W. bancrofti* using immunochromatographic card test (ICT). The study population consisted of 1404 elementary school (ES) students living in Pekalongan District. Overall, 1033 were selected as study subjects. Prevalence survey was also conducted on 436 general population in areas where infected students were found.

**Results:**

The subjects ranged from 7–17 yr old (mean 9.85±1.296) and equally distributed between both sexes. Prevalence of *W. bancrofti* infection was 1.98% in children. Infection was mostly found in older students (12 yr old), male, in 6th grade, but did not differ significantly (*P*=0.129, 0.376, and 0.212 respectively). On the other hand, distribution of infection was significantly different by school (*P*=0.009) and sub-district (*P*=0000). Most of children with LF infection were found in Tirto Sub District. In general population, the prevalence of *W. bancrofti* infection in Tirto was 4.4%. Proportion of infection in males (12.2%) was greater than females (3.8%), with 78.9% of positive cases were in adult over 20 yr old.

**Conclusion:**

Cases of *W. bancrofti* infection exist in Pekalongan District, both in children and adults. Implementation of MDA must be carefully monitored in order to achieve elimination target.

## Introduction

Lymphatic filariasis (LF) is a chronic infectious disease caused by filarial worms *Wuchereria bancrofti*, *Brugia malayi*, and *B. timori* (1,2). The disease is a main community health problem, which mostly affects susceptible people of all ages and sexes (3,4) especially in tropical and subtropical countries (5). About 120 million people in 58 countries are infected globally with an estimated 1.23 billion at risk of infection (6). The disease usually had a very low attention (neglected) in the countries where it is prevalent (7). The manifestations of the disease are noticeably disfiguring (2,3,8), including lymphedema of the limbs, male genitalia (hydrocele), or swollen breast (2).

WHO launched Global Program to Eliminate Lymphatic Filariasis (GPELF) in 2000. The GPELF targeted the elimination of LF as public health problem by 2020 through mass drug administration (MDA) (9). From 2000 to 2013, WHO had delivered more than 5 billion doses of anti-filarial drugs to almost 984 million at-risk individuals worldwide (6). In 2015, Indonesia planned to carry out MDA in 106 LF endemic districts (10). Pekalongan District is an endemic LF area with 62 cases chronic LF, which potentially will continue to increase and spread if not promptly treated.

“Effective monitoring and evaluation are necessary to achieve the goals of LF elimination” (11). After MDA, LF programs must be able to assess whether the prevalence of infection decrease to a level at which transmission is no longer likely to be sustainable (11). Pekalongan District had conducted partial treatment of LF only in its endemic areas (five sub-districts) since 2002. However, data from the District Health Office (DHO) of Pekalongan showed mf rate after the partial MDA (2003–2007) remained high, ranged from 1.15% to 3.90%.

Pekalongan District re-implemented MDA in all sub-districts by 2015. Prior to the MDA, there was no recent data on LF prevalence. Therefore, a baseline survey should be conducted to determine the existence of new infection. LF infection in children is a marker for recent exposure to the parasite (12).

In this study, we conducted a survey prior to the MDA. The study aimed to determine prevalence of *W. bancrofti* infection among elementary school students in endemic sub-districts. The prevalence data will be used as a baseline data because of LF elimination programs will be applied to the whole district.

## Materials and Methods

### Ethics Statement

Ethical approval was obtained from the Committee of Public Health Research Ethics, Diponegoro University (107/EC/FK/2015). Informed consent was acquired from students, in addition to written consent from their parents. Written informed consent was obtained from teachers.

### Study design

This was a descriptive study designed as baseline data to measure prevalence of *W. bancrofti* prior to MDA. Selection of the study site was based on endemicity of LF. Endemicity data was obtained from DHO of Pekalongan. The study was carried out in five LF endemic sub-districts, namely: Buaran, Kedungwuni, Tirto, Wiradesa, and Wonokerto (mf rate in 2007 were 3.9%, 1.27%, 1.15%, 1.40%, and 1.23% respectively). There were a total of 128 elementary schools (ES) in those sub-districts, 28 in Wiradesa, 24 in Wonokerto, 40 in Kedungwuni, 20 in Tirto, and 16 in Buaran. Fifteen ES from those areas were randomly selected.

The protocol for this study was reviewed and approved by the Health Research Ethical Review Committees of the Public Health Faculty, Diponegoro University, Indonesia. The survey was conducted in Sep 2015, a month before the first round of MDA was implemented.

### Subject

**Fig. 1:**
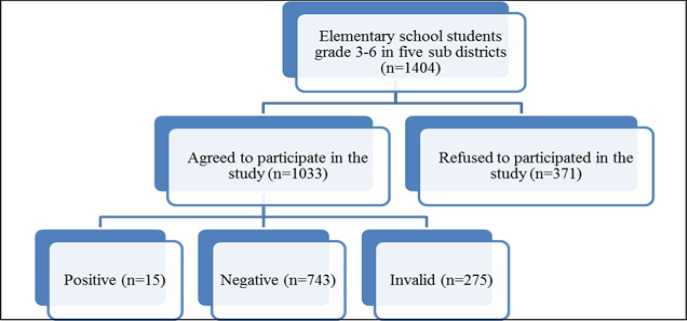
Flow diagram of study subject participation

Inclusion criteria consisted of students from selected ES, in 3^rd^ to 6^th^ grade, and willing to join the study. Study population consisted of 1404 ES students (729 male and 675 female) living in five LF endemic sub-districts in Pekalongan. Of 1404 students, 1033 (98.9%) agreed to undergo CFA examination, as outlined in Fig. 1. Prevalence survey was also conducted in general population of Tirto Sub District, where LF infected students were highest.

### Blood drawing and circulating filarial antigen detection

Circulating filarial antigen (CFA) detection used immunochromatographic test (ICT) from Alere Scarborough Inc., Scarborough - ME USA. A volume of 100 μL blood was drawn by finger prick using microtainer BD blue. The blood was collected in a calibrated capillary tube of each individual.

Blood was then added to the white portion of the sample pad of the card according to the manufacturer protocol. Once the card was closed, the timer was started. The result of the test was read exactly 10 min after closing the card. Pink control (C) and test (T) lines were visible for all valid positive tests, whereas only the control pink line appeared for negative tests.

### Mapping

Mapping of LF used GIS software to produce graphic displays of geographical information. The map showed distribution of LF infection and chronic cases in Pekalongan District. Map of Pekalongan District was obtained from *Dinas Tata Ruang Kabupaten Pekalongan*. Coordinate of LF infection and chronic cases were obtained from GPS.

### Analysis

Data were presented as proportion, mean ± deviation standard, minimum, and maximum. Statistical analysis used Chi-Square to test the difference between positive and negative cases based on sex, grade, school, and sub-district. Mean of age was compared by independent t-test.

## Results

Overall, 1033 students were selected from 15 ES. Among 1033 subjects examined, there were 275 (26.6%) invalid result due to rapid blood clot. The result showed 758 valid results, and 15 subjects were infected by *W. bancrofti*, as outlined in Fig. 1. Prevalence of *W. bancrofti* antigenemia in students was therefore 1.98%. Characteristics of study subjects can be seen in Table 1.

**Table 1:** Characteristics of *Wuchereria bancrofti* antigenemia among elementary school students in Pekalongan District 2015

***Variables***	***CFA***						
	***Positive***		***Negative***		***Invalid***		***P value***
	***n=15***	***%***	***n=743***	***%***	***n=275***	***%***	
Age(yr)							
Mean ± DS	10.27±1.100		9.77±1.256		10.01±1.397		0.129^a^
Median	10		10		10		
Minimum	8		7		7		
Maximum	12		14		17		
Sex							
Male	9	1.7	360	69.5	149	28.8	0.376^b^
Female	6	1.2	383	74.4	126	24.5	
Grade							
Third	1	0.4	195	71.2	78	28.5	0.212^b^
Fourth	5	1.4	272	77.5	74	21.1	
Fifth	8	2.2	255	68.9	107	28.9	
Sixth	1	2,6	21	55.3	16	42.1	

CFA = circulating filarial antigen /DS = deviation standard /

a = Independent t test /

b = Chi-Square test

In general, age of students ranged from 7 to 17 yr old. Mean age of positive subjects was slightly higher compare to negative subjects, yet the difference was not significant (*P*=0.129). The subjects were equally distributed between male and female students. Proportion of positive males was higher than female, although there was no statistical difference (*P*=0.376). Proportion of positive students increased with grade, i.e. 0.4, 1.4, 2.2, and 2.6 respectively from third to sixth grade. No significant difference was found between grades (*P*=0.212).

Distribution of LF in children can be seen in Table 2.

**Table 2:** Distribution of *Wuchereria bancrofti* antigenemia among elementary school students in Pekalongan District 2015

***Variables***	***CFA***						
	***Positive***		***Negative***		***Invalid***		***P value***
	***n=15***	***%***	***n=743***	***%***	***n=275***	***%***	
School							
SDN Kepatihan 1	0	0.0	41	58.6	29	41.4	0.009^b^
SDN Coprayan	0	0.0	54	83.1	11	16.9	
SDN Kemplong	0	0.0	59	78.7	16	21.3	
SDN Simbang Wetan	0	0.0	33	91.7	3	8.3	
SDN Kertijayan 3	0	0.0	39	83.0	8	17.0	
SDN Paweden	2	2.5	55	68.8	23	28.8	
SDN Pekajangan	0	0.0	33	73.3	12	26.7	
SDN Kedungwuni 4	0	0.0	71	78.9	19	21.1	
SD Muhamadiyah 1	0	0.0	60	88.2	8	11.8	
SDN Tegaldowo	5	4.8	86	81.9	14	13.3	
SDN Bondansari 3	0	0.0	20	50.0	20	50.0	
SDN Bebel	0	0.0	47	61.8	29	38.2	
SDN Jeruksari	0	0.0	21	45.7	25	54.3	
SDN Mulyorejo	2	2.4	50	58.1	34	39.5	
SDN Kranding	6	5.8	74	71.2	24	23.1	
Sub Districts							
Wiradesa	0	0.0	120	64.9	65	35.1	0.000^b^
Buaran	2	0.9	181	79.4	45	19.7	
Kedungwuni	0	0.0	164	80.8	39	19.2	
Tirto	13	3.8	231	67.7	97	28.4	
Wonokerto	0	0.0	47	61.8	29	38.2	

CFA = circulating filarial antigen

SDN = *sekolah dasar negeri* = public elementary school

SD = sekolah dasar = *elementary school*

b = Chi-Square test

Cases of LF were only found in two sub-districts, namely Buaran and Tirto Sub Districts.

Proportion of positive CFA in Tirto Sub District was more than 4 times higher than in Buaran Sub District. The school with highest proportion of *W. bancrofti* antigenemia was SDN Kranding, followed by SDN Tegaldowo, SDN Paweden, and SDN Mulyorejo. Three of the schools (SDN Kranding, Tegaldowo, and Mulyorejo) were located in Tirto Sub District. Only one other school was located in Buaran Sub District. Distribution of positive CFA differ significantly according to school (*P*=0.009) and sub district (*P*=0.000).

**Fig. 2:**
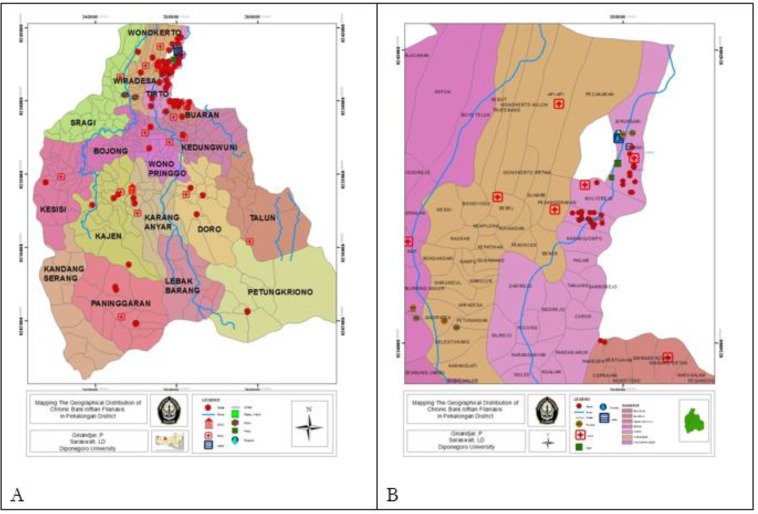
**A.** Geographical distribution of lymphatic filariasis chronic cases in Pekalongan District; **B.** Geographical distribution of *Wuchereria bancrofti* antigenemia in endemic sub districts of Pekalongan District

For further investigation, LF prevalence in general population was examined. The result showed 19 out of 436 subjects were CFA positive. Therefore, prevalence of *W. bancrofti* infection was 4.4%. Mean age was 36.8 yr old in all subjects, while in infected persons the mean age was 39.1 yr old. Proportion of infection in male (8.1%) was higher than female (2.1%). LF cases were also mapped. Fig. 2 shows geographical distribution of chronic and active cases of LF. Most chronic cases were found in Tirto Sub District.

Chronic cases also sporadically spread in other sub districts (Wiradesa, Buaran, Kedungwuni, Wonopringgo, Kajen, Kesesi, Doro, Petungkriono, and Paninggaran).

## Discussion

Study on LF in Pekalongan District is an additional interest to the current effort by DHO of Pekalongan towards LF elimination. This present observation demonstrated LF cases tended to cluster in area of Public Health Center (PHC) Tirto II, especially in village Tegaldowo (SDN Tegaldowo) and Jeruksari (SDN Kranding and Mulyorejo). Tegaldowo and Jeruksari are two neighboring villages. The result, therefore, supports the potential of Tirto Sub District to serve as sentinel site for Transmission Assessment Survey (TAS). TAS is done after five years of eligible MDA program. Selection of sentinel site should be based on mapping of LF endemic areas, i.e. the region with highest cases of LF (13). Tirto is qualified to be a sentinel site when MDA evaluation takes place. Once a sentinel site is selected, it should continue to serve as the sentinel site throughout the program. “Blood surveys at sentinel sites are used to establish the baseline infection level and to monitor the impact of MDA on infection prevalence periodically” (13). Pekalongan District has implemented partial MDA in five endemic sub districts, started in 2002 and ended in 2007 (14). Yet this study found prevalence of LF remains higher than the level of transmission threshold (15,16). According to WHO, after five years of MDA, the expected prevalence of CFA should not exceed 2% (16). With an overall prevalence of 1.98% in children and 4.4% in population, LF remains a public health problem in Pekalongan, especially in Tirto Sub District. Although the prevalence is lower than that of earlier observations (2014) in Indonesia (4.7%) (10), infection in children should receive attention. LF in children is a marker for relatively recent exposure to the parasite (12) and is a sensitive indicator of LF endemicity (17). This study supports the need for targeting children in LF elimination campaign.

“LF prevalence in Indonesia varied from 0.5 to 27.6%. The high rates of LF were found in Maluku, Papua, West Papua, East Nusa Tenggara, and North Maluku” (18). All people at risk are involved in this program (16). The efficacy of six annual rounds of MDA was studied in Alor Island, eastern part of Indonesia. Microfilaria rates in Alor decreased significantly after MDA intervention, from 26% to 0.17%. MDA may be recommended for other parts of Indonesia (19). However, the challenge of MDA was mostly related to the infrastructure of MDA implementation (20) and the compliance with drug administration (21). Therefore, LF elimination in remote area in Indonesia is a major challenge (22). This result showed the prevalence of LF in children increased with age. The similar type of prevalence was also reported from other endemic areas (23–25). Increasing of LF prevalence according to the age in an endemic area correlates with the duration of exposure to the infection (23,26). In this study, the existence of concurrent prevalence of LF both in children and adults was observed in Tirto Sub Districts. Transmission had occurred (27) in the area. Knowledge of LF prevalence in children is very important for understanding the future status of the disease. In this case, the global elimination program decided to protect children from LF (28,29), because children who are close to adults are more exposed to infection (4). This result will help to develop public health strategies for treatment of LF infection in children and to reduce the future disease burden in the adult population.

This study confirmed previous epidemiological studies that proportion of LF was higher in male than female (4,23,25). Higher prevalence in male usually due to the possibility to be exposed to mosquitoes (4). If left untreated, they may experience lymphatic damages and develop hydrocele by around 10–15 yr old, because the parasites seem to prefer the lymphatic of scrotum (30). This study also found LF cases between families, with all family members were infected. The clustering of LF cases possibly due to genetic (31–36) or environmental (34,37) factors. A study in Mauke, Pacific Island revealed a strong association of genetic and LF in population (36). Several other studies in LF endemic areas also showed the infection and microfilariae burden tend to cluster in families, which was mostly due to genetic factor (31–36).

As control effort is implemented, maps of the progress in control can help highlight success and indicate where further effort is required. Mapping of LF was conducted to show the distribution of active LF infection and chronic cases in Pekalongan District (Fig. 2). Both chronic cases in older population and infection in children were mostly found in Tirto Sub District. Tirto is lowland, which located 4 m above sea level. There are two coastal areas in the sub district. Several villages experience tidal inundation and are continuously affected by stagnant water or flood. The existence of stagnant water may serve as breeding places of LF vector Culex quinquefascitus (38). Cx. quinquefascitus was confirmed as LF vector in Samborejo Village, located in Tirto Sub District (39, 40).

During the first three years of partial MDA in Pekalongan District (2002–2005), the coverage increased, i.e. 80.8%, 81.9%, and 90.2% respectively. This impacted in the decrease of mf rate to 0.14% throughout the District in 2007. Tirto and Buaran were two sub-districts with lowest MDA coverage compared to other sub-districts. Consequently, in this study, we found positive cases only in students from Tirto and Buaran. Therefore, the result of this study confirmed the importance of high percentage coverage of MDA to reduce LF transmission.

Information on LF prevalence and associated burdens is necessary to evaluate its public health implication and subsequently plan for control intervention (41). The study showed LF infection both in children and adult. This may serve as baseline prevalence before implementation of MDA. Infected children usually did not show clinical signs. Nevertheless, school-based mass chemotherapy in lower age groups is necessary to prevent clinical manifestations associated with LF infection in the adults later in life (41).

This study described prevalence and geographical distribution of LF in Pekalongan District, and might be used for mapping of population at risk and monitoring the disease.

Limitation of this study related to the selection of study areas. Five sub-districts with high number of chronic LF cases were included in this study. The sub-districts were previously known as LF endemic areas. Therefore, the result cannot be generalized to other non-endemic areas.

## Conclusion

Cases of *W. bancrofti* infection exist in Pekalongan District, both in children (1.98%) and adults (4.4%). LF infection in children is a marker for relatively recent exposure to the parasite and is a sensitive indicator of LF endemicity. Therefore, this result will help to develop public health strategies for treatment of LF infection in children and to reduce the future disease burden in the adult population. Based on geographical distribution, LF infection was found clustering in Tirto Sub-district, located on the northern coast of Central Java. The result supported the potential of Tirto Sub District to serve as sentinel site for Transmission Assessment Survey (TAS). This study may serve as baseline data prior MDA program. Monitoring and evaluation of the program before second round of MDA should be done to measure the prevalence and effect of MDA.

## Ethical considerations

Ethical issues (Including plagiarism, informed consent, misconduct, data fabrication and/or falsification, double publication and/or submission, redundancy, etc.) have been completely observed by the authors.
